# Analyzing the Relationship between Cohort and Case-Control Study Results Based on Model for Multiple Pathogenic Factors

**DOI:** 10.1155/2019/7507043

**Published:** 2019-12-30

**Authors:** Hui Liu

**Affiliations:** Department of Clinical Immunology, Dalian Medical University, Dalian 116044, China

## Abstract

**Objective:**

Although the relative risk from a prospective cohort study is numerically approximate to the odds ratio from a case-control study for a low-probability event, a definite relationship between case-control and cohort studies cannot be confirmed. In this study, we established a different model to determine the relationship between case-control and cohort studies.

**Methods:**

Two analysis models (the cross-sectional model and multiple pathogenic factor model) were established. Incidences in both the exposure group and the nonexposure group in a cohort study were compared with the frequency of the observed factor in each group (diseased and nondiseased) in a case-control study.

**Results:**

The relationship between the results of a case-control study and a cohort study is as follows: *Pe*=(*Pd∗m*)/(*Pc∗m*)/(*Pd∗m*)/(*Pn*=(*m*)/(*∗PdPc∗m*)/(*Pd∗m*)/(*Pe* and *Pn* represent the incidence in the exposed group and nonexposed group, respectively, from the cohort study, while *Pd* and *Pc* represent the observed frequencies in the disease group and the control group, respectively, for the case-control study; finally, *m*)/(

**Conclusions:**

There is a definite relationship between the results of case-control and cohort studies assessing the same exposure. The outcomes of case-control studies can be translated into cohort study data.

## 1. Introduction

Currently, the pathogenesis of most diseases is related to interactions among extrinsic and intrinsic risk factors. Interactions between multiple factors are complex and difficult to tease out [1–3]. To study the correlation between a specific factor and a disease, cohort studies (including prospective and retrospective) are considered to be the more robust form of scientific evidence in the hierarchy of evidence [4–6]. In a cohort study, a suspected risk factor should be considered an exposure factor, and the exposed and unexposed subjects should be observed until they develop the outcome of interest. Furthermore, the ratio of the incidences of the two groups (relative risk or RR) and difference in the incidences between the two groups (absolute risk or AR) are used to evaluate the role that this factor has in the pathogenesis of the disease [7–9]. Given that pathogenesis precedes disease in the cohort design, the cohort study has a stronger ability to test hypotheses and generally confirms the association, if it exists [4–6]. However, cohort studies are limited for diseases with low incidence. For diseases with low incidence, long-term observation of large populations is necessary; therefore, the cost of materials and time is high. Furthermore, increased loss to follow-up due to long-term observation affects the quality of research so that cohort studies are not possible in many disease studies.

As an alternative, case-control studies are conducted in most studies of aetiological factors associated with infrequent disease [10]. In case-control studies, the potential relationship between a suspected risk factor or an attribute and the disease is examined by comparing frequencies of this factor or attribute in the diseased and nondiseased subjects. Compared with prospective cohort studies, they tend to be less costly and shorter in duration. However, the measure of association, the odds ratio (OR; the ratio of the odds of A in the presence of B and the odds of A without the presence of B) from a case-control study is an inferior measure of association compared to relative risk (RR) [11–13]. This type of research design is chronologically reversed in which we assess causal factors from outcome. It is a controversial issue when drawing conclusions about causality when using the case-control design. However, in many scenarios, case-control studies are often the only realistic choice of research methodology. Therefore, it is necessary to further explore the relationship between the two methods.

It has analyzed that relationship between cohort and case-control study results according to cross-sectional study and found that the RR associated with a pathogenic factor in a prospective cohort study is numerically approximate to the OR from a case-control study when contracting the disease of interest is a low-probability event [10–12]. However, the incidence in nonexposed groups in cohort studies cannot be obtained in such conditions, and a definite relationship between the results of case-control and cohort studies was difficult to establish. Although a rectifying formula was used to calculate the incidence in the nonexposed group [14], it cannot be confirmed that the result from the cross-sectional model is appropriate for that from the multiple factor model; therefore, the original data from case-control studies cannot be transformed into cohort study findings.

In this study, a model for multiple pathogenic factors was established and a definite relationship between the results of case-control and cohort studies was inferred. The outcomes of case-control studies can be translated into cohort study data.

## 2. Methods

### 2.1. A Definite Relationship between the Binary Outcomes of Case-Control and Cohort Studies Based on a Cross-Sectional Model

A cross-sectional model was established according to the literature [14]. We assumed that the frequencies of a cross-sectional outcome were distributed as shown in Table 1.

Now,

(1) $$\begin{array}{c} \frac{Pc}{Pd}=\frac{b/a}{\left(b+d\right)/\left(a+c\right)}=\frac{\left(1-Pe\right)/Pe}{\left(1-m\right)/m}=\frac{\left(1/Pe\right)-1}{\left(1-m\right)/m}. \end{array}$$

Solving for *Pe*, this becomes

(2) $$\begin{array}{c} \frac{m*Pd}{m*Pd+\left(1-m\right)*Pc}=Pe. \end{array}$$

Similarly,

(3) $$\begin{array}{c} \frac{1-Pc}{1-Pd}=\frac{d/c}{\left(b+d\right)/\left(a+c\right)}=\frac{\left(1+Pn\right)/Pn}{\left(1-m\right)/m}=\frac{\left(1/Pn\right)-1}{\left(1-m\right)/m}. \end{array}$$

Solving for *Pn*, this becomes

(4) $$\begin{array}{c} \frac{m*\left(1-Pd\right)}{1-m*Pd-\left(1-m\right)*Pc}=Pn. \end{array}$$

### 2.2. Multiple Pathogenic Factor Model considering Morbidity

**Figure 1:**
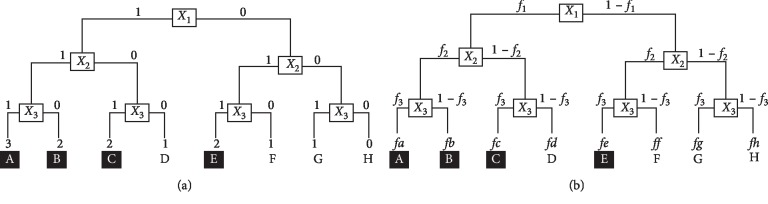
The frequency distribution of three pathogenic factors in the model. *X*1, *X*2, and *X*3 represent the pathogenic factors. A, B, C, and E represent the combinations of these factors that correspond to the onset of the disease, whereas D, F, G, and H represent the combinations for which the disease does not occur. “*f*” represents a frequency distribution of risk factors.

The model for multiple pathogenic factors was built according to the literature [15]. This model proposed that there are 3 independent risk factors: *X*1, *X*2, and *X*3, all related to a disease and that any two of these factors together (superimposed manner, regardless of interaction) could induce the disease, whereas one factor alone could not induce the disease. The model is detailed in Figure 1.

We proposed that the frequencies of *X*1, *X*2, and *X*3 are *f*1, *f*2, and *f*3, respectively; then, the frequencies of A–H could be calculated (see Figure 1(b)).

Thus, frequencies D (*fd*) and frequencies E (*fe*) can be expressed in the following examples:

(5) $$\begin{array}{c} fd=f_{1}*\left(1-f_{2}\right)*\left(1-f_{3}\right),\\ fe=\left(1-f_{1}\right)*f_{2}*f_{3}. \end{array}$$

#### 2.2.1. The Calculation of the Incidence in a Cohort Study

Using the observation of the *X*1 pathogenic factor as an example and given that any two of the factors together could induce disease (see Figure 1), the incidence of the disease in the exposed group with the *X*1 factor (*Pe*) is

(6) $$\begin{array}{c} Pe=\frac{fa+fb+fc}{f_{1}}=\frac{f_{1}-fd}{f_{1}}=1-\frac{fd}{f_{1}}, \end{array}$$

and the incidence in the nonexposed group without the *X*1 factor (*Pn*) is

(7) $$\begin{array}{c} Pn=\frac{fe}{1-f_{1}}. \end{array}$$

#### 2.2.2. The Calculation of the Frequency of Observed Factors in Each Group in a Case-Control Study

Using Figure 1 as an example, the frequency of individuals with the *X*1 factor in the disease group (*Pd*) is

(8) $$\begin{array}{c} Pd=\frac{fa+fb+fc}{fa+fb+fc+fe}=\frac{f_{1}-fd}{f_{1}-fd+fe}, \end{array}$$

and the frequency of individuals with the *X*1 factor in the control group (nondisease group) (*Pc*) is

(9) $$\begin{array}{c} Pc=\frac{fd}{fd+ff+fg+fh}=\frac{fd}{fd+\left(1-f_{1}\right)-fe}=\frac{fd}{1-f_{1}-fe+fd}. \end{array}$$

#### 2.2.3. Comparison of the Results of the Case-Control Study with Those of the Cohort Study

We propose that the incidence of a disease in the total population is *m*:

(10) $$\begin{array}{c} m=fa+fb+fc+fe=f_{1}-fd+fe. \end{array}$$

Because

(11) $$\begin{array}{c} Pd=\frac{f_{1}-fd}{f_{1}-fd+fe},\\ Pc=\frac{fd}{1-f_{1}-fe+fd}. \end{array}$$

The values of *Pd* and *Pc* are derived from the case-control study; *m* is derived from the investigation; thus, *f*_1_, *fe*, and *fd* can be obtained from *m*, *Pd*, and *Pc* by calculating the solution of the equation.

This is because

(12) $$\begin{array}{c} Pe=1-\frac{fd}{f_{1}},\\ Pn=\frac{fe}{1-f_{1}}. \end{array}$$

Conversely, *Pe* and *Pn* can be calculated from *f*_1_, *fe*, and *fd*.

### 2.3. Comparing Differences between the Two Groups in Case-Control and Cohort Studies

The relationship of the difference between the disease and control groups in the case-control study with that between the exposed and nonexposed groups in the cohort study was observed based on the obtained formula. We plotted the scatter diagram using the difference between *Pd* and *Pc* in the case-control study as *X*-axis and the difference between *Pe* and *Pn* in the case-control study as *Y*-axis. The incidence with 0.05, 0.01, and 0.001 was, respectively, considered as the incidence of a disease in the total population (*m*) to set up scatter diagrams.

## 3. Results and Discussion

The relationship between the results of a case-control study and those of a cohort study was established based on a model for multiple pathogenic factors as follows:

(13) $$\begin{array}{c} Pe=\frac{Pd*m}{Pc*\left(1-m\right)+Pd*m},\\ Pn=\frac{m*\left(1-Pd\right)}{1-Pc*\left(1-m\right)-Pd*m}, \end{array}$$

where *Pe* and *Pn* represent the incidences in the exposed group and the nonexposed group, respectively, in a cohort study; *Pd* and *Pc* represent the frequencies of the observed factor in the disease group and in the control group, respectively, in a case-control study; and *m* represents the incidence in the total population.

The cohort exposed group (*Pe*) and nonexposed group (*Pn*) derived from the case-control study according to the multiple pathogenic factor model were the same with respect to the data from the cross-sectional model, confirming that these models were established correctly.

In the case of very low incidence (*m* = 0),

(14) $$\begin{array}{c} \text{RR}=\frac{Pe}{Pn}=\frac{Pd*\left[1-Pc*\left(1-0\right)-Pd*0\right]}{\left[Pc*\left(1-0\right)+Pd*0\right]*\left(1-Pd\right)}=\frac{Pd*\left(1-Pc\right)}{Pc*\left(1-Pd\right)}, \end{array}$$

where (*Pd∗*(1 − *Pc*))/(*Pc∗*(1 − *Pd*)) is the odds ratio (OR) in the case-control study [11–13]. This result is also consistent with general suggestions [11–13], further confirming that the model was correct from another angle.

In most cases, the distribution of risk factors was high, such as ageing, social, and environment factors or life style; therefore, OR is not suitable for evaluation of the degree of risk at this time. For example, if we consider the constituent ratio of death from lung cancer was 0.083 and the frequency of short tandem repeats (STRs) was also high, the OR value was not equal to the RR value [13]. In this study, where the case-control design was adopted, cancer risk was simultaneously evaluated from many STRs; loss to follow-up in the cohort study could be avoided. In this scenario, the formula can be used to convert the data from the case-control study into the data of the prospective study to calculate the RR.

In a case-control study, the most important drawback is that information from the case-control study is believed to be difficult in the quantitative assessment of risk factors. Therefore, cohort studies are generally the basis for understanding multifactorial pathogenesis. Given that most research data are derived from case-control studies, case-control studies should be compared with cohort studies. In the present study, a formula for comparing the results of case-control and cohort studies was developed for translating data from case-control studies into data associated with prospective cohort studies, making it possible to replace cohort studies with high-quality case-control studies.

One of the biggest benefits of case-control studies in a medical context is their ability to address infrequent diseases. As we were able to demonstrate a definite relationship between the results of the case-control and cohort studies, the outcomes of case-control studies can be translated into the results of cohort studies. Therefore, the efficiency and efficacy of observational research could be increased.

**Figure 2:**
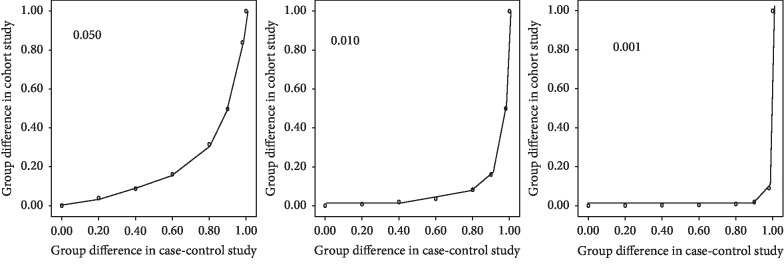
A scatter diagram of the relationship between the difference of the two groups in the case-control study and the difference of the two groups in the cohort study. 0.050, 0.010, and 0.001 represent the incidence of disease in the total population.

We also observed the relationship of the difference between the two groups in the case-control study with that between the two groups in the cohort study based on the obtained formula as shown in Figure 2 to clarify how risk in the case-control study can relate to the cohort study, in a pragmatic manner.

The plotted scatter diagrams revealed that the inflection points are 0.90 and 0.49 for the 0.050 model (*m* = 0.05), 0.98 and 0.050 for the 0.010 model (*m* = 0.01), and 0.98 and 0.009 for the 0.001 model (*m* = 0.001). When the difference between the two groups in the case-control study was >0.90 to reach 0.5 difference between the two groups in the cohort study for a 0.05 incidence in the total population (*m* = 0.05), >0.98 to reach 0.5 difference between the two groups in the cohort study (*m* = 0.01), and >0.99 to reach 0.5 difference between the two groups in the cohort study (*m* = 0.001). Obviously, the case-control study may lead to overestimations of effects of risk factors. It might be misleading to pay attention only to the results of case-control studies.

## Figures and Tables

**Table 1:** Frequency distribution of individuals in each group for a cross-sectional outcome.

Group	Disease (case)	Nondisease (control)	Risk	Odds
Exposed (factor)	*a*	*b*	*Pe* = *a*/(*a* + *b*)	*Pe*/(1 − *Pe*) = *a*/*b*
Nonexposed (control)	*c*	*d*	*Pn* = *c*/(*c* + *d*)	*Pn*/(1 − *Pn*) = *c*/*d*
Total	*a* + *c*	*b* + *d*	*m* = (*a* + *c*)/(*a* + *b* + *c* + *d*)	*m*/(1 − *m*) = (*a* + *c*)/(*b* + *d*)
Obs. frequencies	*Pd* = *a*/(*a* + *c*)	*Pc* = *b*/(*b* + *d*)	—	—

*a*, *b*, *c*, and *d*: frequency of individuals in each group; *Pd* and *Pc*: frequency of individuals with the related factor in the disease group and that in the nondisease group; *Pe* and *Pn*: incidence of the exposed group and that of the nonexposed group; m: incidence of a disease in the total population.

## Data Availability

The data used to support the findings of this study are available from the corresponding author upon request.
